# Prevalence and genetic characterization of porcine circovirus type 2, 3 and 4 in the upper Northern region of Thailand

**DOI:** 10.1007/s11259-025-10786-w

**Published:** 2025-06-10

**Authors:** Wichittra Anukool, Panuwat Yamsakul

**Affiliations:** 1 Veterinary Research and Development Center (Upper Northern Region, Department of Livestock Development, 221 M.6 Wiang Tan sub-district, Hang Chat district, 52190 Lampang, Thailand; 2 School of Veterinary Medicine, Faculty of Veterinary Medicine, Chiang Mai University, 155 M.2, Maehae sub-district, Mueang district, Chiang Mai, 50100 Thailand

**Keywords:** Porcine circovirus, Prevalence, Genetic characterization, Upper Northern region of Thailand

## Abstract

Porcine circovirus (PCV) poses challenges to swine health and production worldwide, with PCV2, PCV3, and may be PCV4 contributing to porcine circovirus-associated diseases (PCVAD). This study investigated the prevalence and genetic diversity of PCV2, PCV3, and PCV4 in fattening pigs from 42 farms in Chiang Mai, Lamphun, and Lampang Provinces, Thailand. A total of 396 blood samples were tested using real-time PCR, and positive samples were selected for partial capsid (cap) gene sequencing. Real-time PCR detected PCV2 in 22.73% of samples and PCV3 in 4.29%, with co-infection found in 2.78%. PCV4 was not detected. Although the Chi-square test (𝜒² = 0.937, *P* > 0.05) showed no significant association between PCV2 and PCV3 infections and the odds ratio for PCV2 and PCV3 infection was 1.92 (95% CI: 0.69–5.33, *P* > 0.05), and the relative risk (RR) was 1.85, but suggesting a potential link between PCV2 infection and increased PCV3 infection. Genetic analysis identified all PCV2-positive samples as PCV2d and PCV3-positive samples belonged to clades 3a and 3b, with geographical clustering. Lamphun samples contained only 3a, while both 3a and 3b were found in Lampang. These findings highlight the dominance of PCV2d and the need for targeted vaccination. While PCV2 vaccines are available, commercial vaccines for PCV3 and PCV4 remain unavailable, emphasizing the importance of ongoing surveillance and research for effective disease control in Thai swine farms.

## Introduction

Porcine circovirus (PCV) is a single-stranded DNA virus in the Circoviridae family that causes economic losses in the swine industry worldwide. Four types of PCV (PCV1–PCV4) have been identified. Among them, PCV2 is the most pathogenic, associated with postweaning multisystemic wasting syndrome (PMWS), porcine dermatitis and nephropathy syndrome (PDNS), and porcine respiratory disease complex (PRDC) (Opriessnig et al. 2020). PCV2 is classified into nine genotypes (PCV2a–PCV2i), while PCV3 is grouped into three clades (PCV3a–PCV3c) with further subdivisions (Maity et al. 2023), and PCV4 into two groups (PCV4a and PCV4b) (Xu et al. 2022). PCV3 and PCV4 have been detected in pigs with PDNS-like symptoms and systemic inflammation (Zhang et al. 2020; Sirisereewan et al. 2023). Transmission occurs via oro-nasal contact, secretions, and vertical pathways. PCVs have also been reported in wild boars, rodents, cattle, and dogs (Tan et al. 2021). Unlike PCV2, PCV3 and PCV4 can be found in pigs across all age groups, in both healthy and diseased animals, often as co-infections. Understanding their epidemiological patterns is crucial for improving disease surveillance and control.

The genome sizes of PCV2, PCV3, and PCV4 are approximately 1767–1777, 1999–2001, and 1770 nucleotides, respectively (Maity et al. 2023). Each virus contains two major open reading frames (ORFs): ORF1 (rep gene) encoding the replication protein, and ORF2 (cap gene) encoding the capsid protein (Opriessnig et al. 2020). Several detection methods are available, including ISH, IHC, and ELISA. However, PCR-based nucleic acid detection is the most used approach, targeting either the rep or cap gene (Tan et al. 2021). For phylogenetic analysis, the cap gene is preferred due to its role as the main antigenic protein and relatively conserved sequence compared to the rep gene, which is more variable under selective pressure (Olvera et al. 2007). Moreover, PCVs are classified into four types: PCV1, PCV2, PCV3, and PCV4 (Opriessnig et al. 2020). Among them, PCV2 is the most concerning due to its global spread and disease association. It likely emerged around 1966 in the US and Germany, and multiple genotypes have since been identified, with genotype shifts linked to disease severity and vaccine response (Franzo and Segalés 2018). In Thailand, PCV2 prevalence varies by region and age group. PCV3 was first reported in the US in 2015 and subsequently detected in China, South Korea, Italy, Brazil, and India (Phan et al. 2016). Thai studies since 2018 show PCV3 prevalence ranging from 28 to 60% (Sirisereewan et al. 2023). PCV4, discovered in China’s Hunan Province, has also been found in Korea and Thailand, though at a low prevalence (~ 0.4%) (Zhang et al. 2020; Sirisereewan et al. 2023). However, data from Thailand’s Upper Northern Region remain scarce (Visuthsak et al. 2021), despite its mix of smallholder and commercial farms. Continued molecular surveillance is crucial for monitoring viral evolution, recombination, and immune escape (Opriessnig et al. 2020).

Therefore, this study aims to determine the prevalence of the PCV2, PCV3 and PCV4 in swine farms across the Upper Northern Region of Thailand. Additionally, we will investigate the genetic characteristics of each viral type to provide surveillance data for veterinarians and swine farm managers in the Upper Northern Region.

## Materials and methods

### Sample collection

A total of 396 blood samples of healthy fattening pigs (16–20 weeks of age) were collected from 42 fattening pig farms located in the Upper Northern Region of Thailand during January 2025. The sample size was calculated using the G-power™ software with 0.3 effect size, 0.05 significance level, and 0.8 1-β, based on the swine population density across three provinces: Chiang Mai, Lamphun, and Lampang Provinces. A simple random sampling (SRS) method was used to select the farms so that each farm had an equal probability of being included in the study. This approach minimizes selection bias and enhances the representativeness of the sample. The distribution of samples is presented in Table 1. All samples were submitted to the Animal Disease Diagnostic Laboratory of Veterinary Research and Development Center (Upper Northern Region) in Lampang Province.

**Table 1:** The distribution of blood samples collected in the study

Provinces	Number of farms	Number of samples
Chiang Mai	6	63
Lamphun	26	233
Lampang	10	100
Total	42	396

*** The study did not involve any animal experiments. All samples were submitted to the Veterinary Research and Development Center (Upper Northern Region), Department of Livestock Development for diagnostic purposes. The permission for data usage was granted by each farm and approved by the Animal Care and Use Committee of Nation Institute of Animal Health, Thailand, approval number is EA-001/68 (R).

### Viral DNA extraction and detection of PCV2, PCV3 and PCV4

The Viral DNA was extracted from 200 microliters (µl) of blood samples using the QIAamp DNA Blood Mini Kit (Qiagen^®^, Germany) following the manufacturer’s protocol. The extracted viral DNA was subjected to real-time PCR analysis using the Kylt^®^ PCV-2 and Kylt^®^ PCV-3 Test Kits (Sangroup Biotech, Germany). The PCR conditions were initial denaturation at 95 °C for 10 minutes, followed by 42 cycles of 95 °C for 15 seconds and 60 °C for 1 minute. For the PCV4 detection, real-time PCR was performed according to Sirisereewan et al. (2023), targeting the *replicase (rep)* gene using primers (F) 5’-AAAGCGCAGCGACCTTAAAG-3’, (R) 5’-CACGGGCCACTTCACTCATT-3’ and probe 5’-ROX-CTGTGGCCGCCCTGAATGCC-BHQ2-3’. The reaction conditions included initial denaturation at 95 °C for 10 min, followed by 45 cycles of 95 °C for 15 s and 60 °C for 30 s. The LightCycler 480 Probes Master (Roche^®^, Germany) was used with a final primer concentration of 0.5 micromolar (µM), using the Quanstudio^®^ 5 Real-time PCR System (ThermoFisher, USA). Then, a positive sample per farm for each PCV type was selected for genetic sequencing analysis.

### Capsid gene amplification and genetic sequencing

Representative samples that tested positive for each PCV type from different areas were selected for partial *capsid (cap)* gene amplification. The primers used are shown in Table 2. Amplification was performed using GoTaq^®^ Green Master Mix (Promega, USA) in 25 µl reaction volumes.

**Table 2:** Nucleotide sequences of primers used for *capsid (cap)* gene amplification in this study

PCV type	Primer sequences (5’−3’)		Size of the product (bp)	References
PCV2	PCV2-939 PCV2-33	GCC GAG GTG CTG CCG CT CAG TTC GTC ACC CTT TCC CC	863	Yang et al. (2008)
PCV3	PCV3-649–1 F PCV3-649-1R	TTACTTAGAGAACGGACTTGTAACG AAATGAGACACAGAGCTATATTCAG	649	Visuthsak et al. (2021)
PCV4	PCV4-904 F PCV4-1745R	TGAGGGAGGATGGGCAGTTGTATG CACCACCCACAGATGCCAATCA	842	Zhang et al. (2020)

The PCR products were purified using the QIAquick^®^ PCR Purification Kit (QIAGEN, Germany). To confirm the sequences, partial *cap* gene fragments were subcloned into the pGEM^®^-T Easy Vector System (Promega, USA) following the manufacturer’s instructions. They were then sequenced using an automated DNA sequencer based on the Sanger method.

### Phylogenetic analysis

Nucleotide sequences of the PCV2, PCV3 and PCV4 were analyzed and aligned using ChromasPro version 2.1.10 (Technelysium Pty. Ltd.). The sequences were compared with those obtained from GenBank *(*https://www.ncbi.nlm.nih.gov/nucleotide/*)* using the multiple sequence comparison by log-expectation (MUSCLE) alignment method (Edgar 2004). Phylogenetic trees were constructed using the neighbor-joining (NJ) algorithm with the p-distance model and 1,000 bootstrap replicates in Molecular Evolutionary Genetics Analysis (MEGA) version 11.0.13 (Tamura et al. 2021).

### Geographical distribution analysis

The geographical distribution of PCV2, PCV3 and PCV4 was analyzed using areal interpolation and dasymetric methods (Briggs et al. 2007), correlating viral genetic characteristics with their spatial distribution patterns. For the geographical distribution analysis, we utilized GIS software (e.g., QGIS) to perform areal interpolation and dasymetric mapping.

### Statistical analysis

The prevalence of PCV2, PCV3 and PCV4 was calculated at the provincial level based on real-time PCR results using descriptive statistics. The analytical statistics were also applied to evaluate the associations between infections of the PCV2, 3 and 4 using contingency tables and tested with the Chi-square test. Additionally, the odd ratio (OR) and the relative risk (RR) ratios were calculated to compare the probability of infection among different PCV types by R version 4.4.1.

## Results

The real-time PCR testing revealed 90 positive samples for the PCV2 and 17 positive samples for the PCV3, representing 22.73% and 4.29% prevalence, respectively. The PCV4 was not detected in any of the tested samples. Moreover, 11 positive samples co-infected with PCV2 and PCV3, representing 2.78%, highlight the importance of investigating their epidemiological relationship (Table 3).

**Table 3:** The detection of Porcine circovirus types 2, 3, and 4 in fattening pigs from upper Northern Thailand using the real-time PCR

PCV type	Positive samples	Individual prevalence (%)	Positive farms	Farm prevalence (%)
PCV2	90	22.73	16	38.10
PCV3	17	4.29	7	16.67
PCV4	0	N/A	0	N/A
PCV2 with PCV3	11	2.78	5	11.90

Co-infection with PCV2 and PCV3 was identified in certain samples (Table 4). Statistical analysis using contingency tables revealed no significant association between the two infections (χ² = 0.937, *P* = 0.333). The odds ratio (1.92; 95% CI: 0.69–5.33) and relative risk (1.85; 95% CI: 0.71–4.88) were not statistically significant (*P* > 0.05). Although these values suggest a possible trend toward increased PCV3 detection in PCV2-positive farms, the confidence intervals include 1, indicating no conclusive association. Further studies with larger sample sizes are recommended to clarify the epidemiological relationship between PCV2 and PCV3.

**Table 4:** The contingency table between PCV2 and PCV3 infections

Samples	PCV3 infected	PCV3 non-infected	Total
PCV2-infected	6	84	90
PCV2 non-infected	11	295	306
Total	17	379	396

**Fig. 1:**
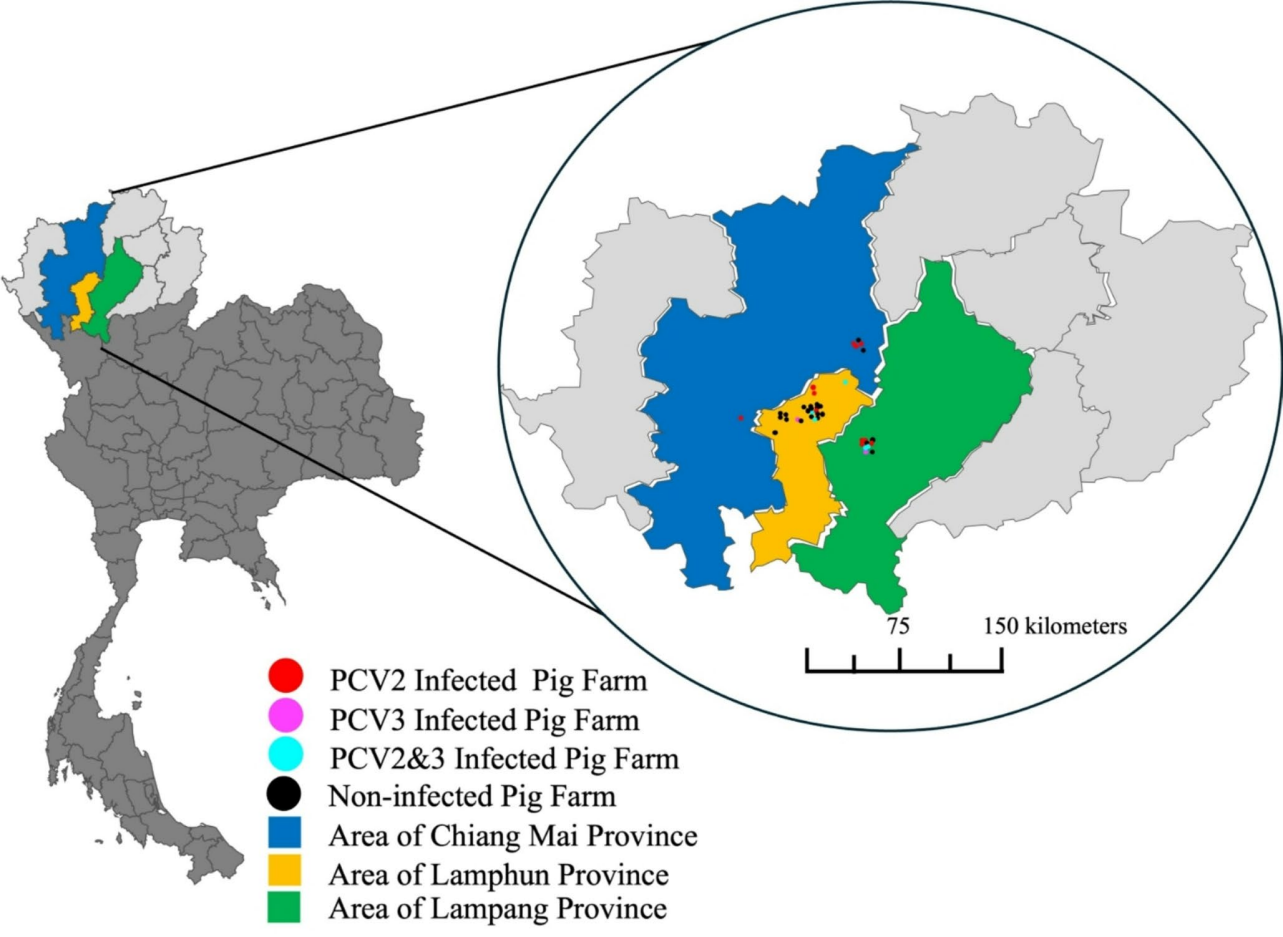
The geographical distribution of collected blood samples and infected fattening pig farms in the Upper Northern Thailand

**Fig. 2:**
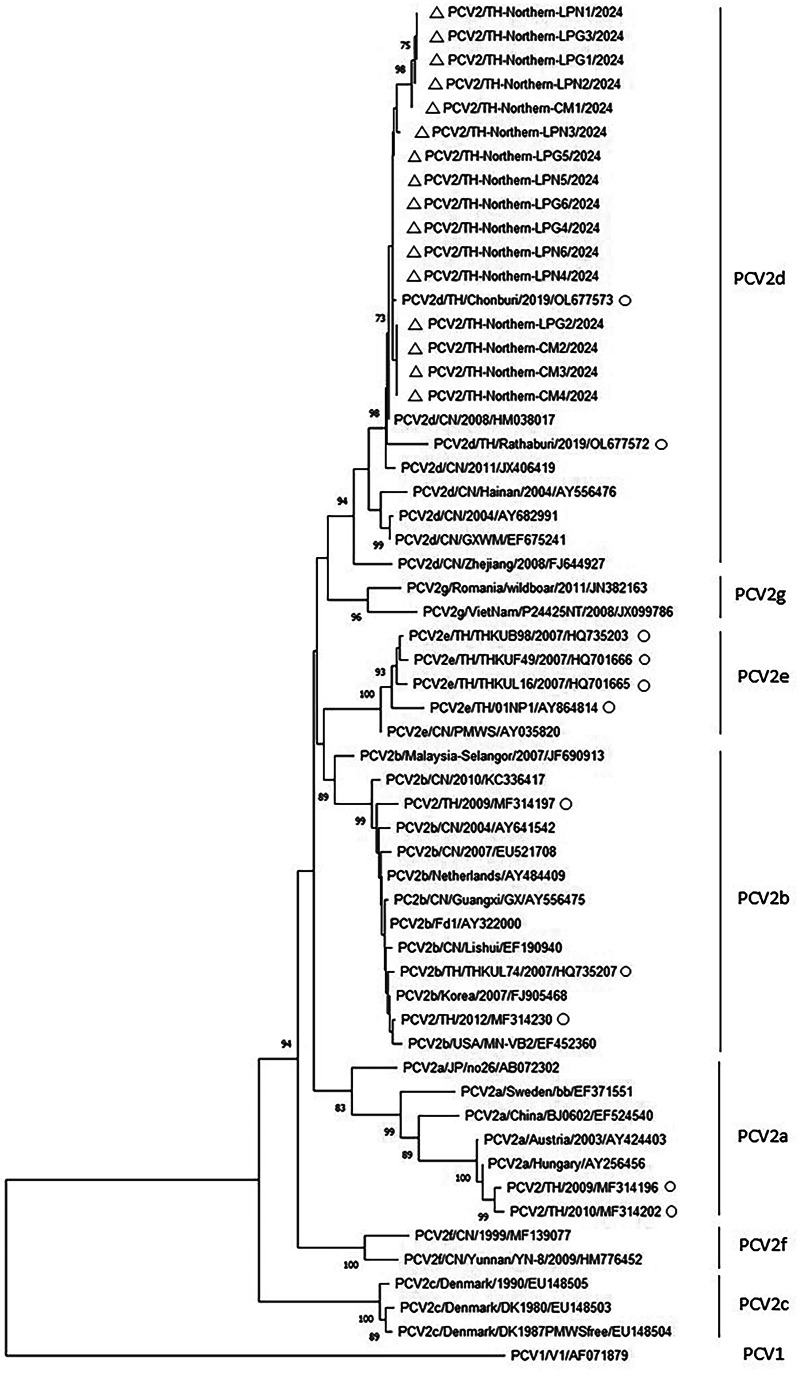
Phylogenetic analysis with partial genome of ORF2 (*cap*) of PCV2 by the neighbor-joining method with bootstrap analysis of 1,000 replicates. Triangles (Δ) represented nucleotide sequences in this study. Circles (O) represented Thai PCV2 sequences retrieved from GenBank. Accession numbers were presented in the latter of sequences’ name

The geographical distribution of the PCV2 and PCV3 prevalence in the Upper Northern Region is illustrated in the map (Fig. 1). In this study, 16 PCV2-positive and 7 PCV3-positive samples, selected as farm representatives from the real-time PCR testing, underwent genetic sequencing. All PCV2 samples were classified as PCV2 d (Fig. 2), showing close genetic relationships with strains from Chonburi and Ratchaburi Provinces, Thailand in 2019 (Accession no. OL677573 and OL677572, respectively) and strains from China (HM038017).

**Fig. 3:**
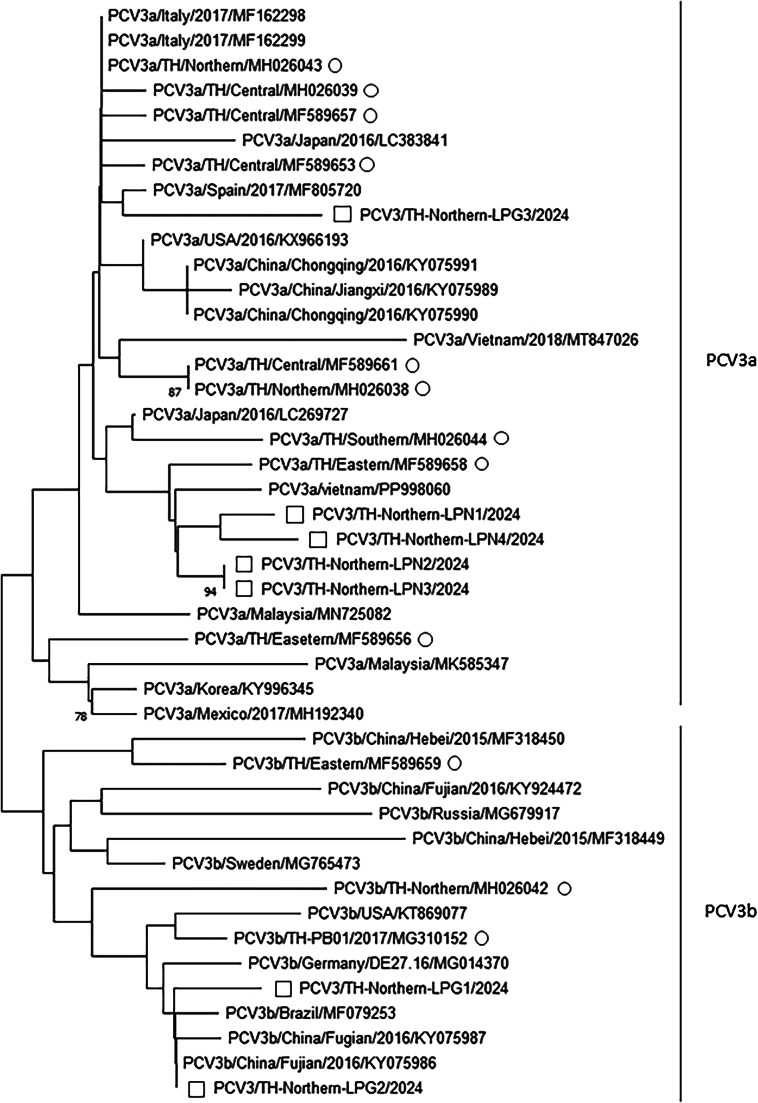
Phylogenetic analysis with partial genome of ORF2 (*cap*) of PCV3 by the neighbor-joining method with the p-distance parameter associated with 1,000 replicates of bootstrap test. Squares (□) represented nucleotide sequences in this study. Circles (O) represented Thai PCV3 sequences retrieved from GenBank. Accession numbers were presented in the latter of sequences’ name

Regarding PCV3, the samples contained both PCV3a and PCV3b clades. The Lamphun province samples (LPN1-LPN4) exclusively belonged to PCV3a, while the Lampang province samples included both 3a (LPG3) and 3b (LPG1-LPG2) (Fig. 3). The clade analysis of PCV3a revealed close genetic relationships among the LPN1-LPN4 strains, clustering with Vietnamese isolates and the Eastern Thai strains (Accession no. MF589658) from 2014.

## Discussion

The porcine circovirus (PCV) has significant implications for the global swine industry, particularly the PCV2, which is currently prevalent in swine farms. However, there has been an increasing number of reports documenting the emergence of the PCV3 and PCV4 in swine operations.

The PCV2 was first reported in Thailand in 1998 in 7–9-week-old pigs (Tantilertcharoen et al. 1999), although retrospective analysis indicated its presence since 1993. The virus remains endemic, with recent studies reporting animal- and farm-level prevalence rates of 54.2% and 81.4%, respectively (Sirisereewan et al. 2023), comparable to the present study’s farm-level prevalence of 38.10%. PCV3 was initially identified in the United States in 2015 through metagenomic sequencing (Palinski et al. 2017), but retrospective findings suggest global circulation since the early 1990 s (Maity et al. 2023). In Thailand, PCV3 was first reported in fattening pigs, with detection in preserved tissues from 2006, supporting widespread and prolonged circulation. However, its pathogenic role remains inconclusive, with inconsistent associations to porcine respiratory disease complex (PRDC) and postweaning multisystemic wasting syndrome (PMWS) (Kedkovid et al. 2018; Visuthsak et al. 2021). PCV4 was not detected in this study, consistent with prior findings from Chiang Mai Province (Sirisereewan et al. 2023), although sporadic detection in other provinces suggests low prevalence. Notably, the aim of this study was not to correlate infection with clinical signs, but to assess farm-level infection status, particularly subclinical infections. Such infections, despite the absence of overt symptoms, may adversely impact productivity and serve as hidden reservoirs (Kekarainen and Segalés 2012; Opriessnig et al. 2020). Co-infection with PCV2 and PCV3 is likely driven by viral persistence, immunosuppression, and environmental factors, including vector transmission via insects (Blunt et al. 2011; Ha et al. 2020; Maity et al. 2023). Clinical manifestations alone are insufficient for diagnosis; thus, laboratory confirmation and clinical correlation are necessary for accurate assessment (Sukmak et al. 2019; Visuthsak et al. 2021).

Although, the Chi-square test (𝜒²) showed an association value of 0.937 between PCV2 and PCV3 infection, which was not statistically significant (*P* > 0.05) that mean there is not enough evidence to confirm a significant statistical association between PCV2 and PCV3 infections. This implies that, based on the available data, the occurrence of these two infections may not be directly related. The odd ration between PCV2 and PCV3 is 1.92 (95% Confidence Interval (CI) is 0.69–5.33 and *P* > 0.05) that mean PCV2-infected farms had a 1.92-fold higher likelihood of PCV3 infection than non-PCV2-infected farms. As this study is a cross-sectional study, the odds ratio is typically used in epidemiological analysis. However, we selected the relative risk (RR) addition, as the prevalence of PCV3 infection in our study was low, making the rare outcome assumption valid. Given this low prevalence, RR is more suitable for interpretation than the odds ratio. The relative risk (RR) of PCV3 infection in the PCV2-infected group compared to the non-PCV2-infected group was 1.85, suggesting that PCV2 infection may be associated with an increased risk of PCV3 infection. This RR suggests a potential increase in PCV3 infection risk in PCV2-positive farms, despite not reaching statistical significance. These findings may imply a biological link or shared risk factors between PCV2 and PCV3 infections, warranting further investigation.

The genetic characteristics of PCVs can be classified into subtypes. Previous reports of PCV2 in Thailand identified subtypes a, b, d, and e, with this study confirming that all samples belonged to subtype d. PCV2 d predominates at both the national and regional levels (Jittimanee et al. 2011; Thangthamniyom et al. 2017; Sirisereewan et al. 2023). For PCV3, both PCV3a and PCV3b clades are present in Thailand (Sukmak et al. 2019). Initial genetic characterizations from 2018 showed relationships with strains from South Korea, the United States, China, and Brazil (Kedkovid et al. 2018). Studies have identified PCV3 in Thailand since 2006 in both symptomatic and asymptomatic pigs, with strains related to Thai and Korean isolates. Molecular evolution analysis indicated no geographical correlation (Sukmak et al. 2019). A 2021 study found exclusively PCV3a related to Brazilian, Korean, and Italian strains, suggesting low genetic variability or continuous viral circulation in Thailand (Visuthsak et al. 2021). In this study, the LPG3 sample showed closer genetic similarity to Spanish isolates and strains from Northern Thailand (Sukmak et al. 2019). PCV3b exhibited proximity to Chinese isolates from 2016 and Thai strains from 2017, suggesting regional variants or recent emergence.

PCV disease prevention primarily relies on vaccination, with commercial vaccines targeting PCV2a. These vaccines offer cross-protection against PCV2b and 2 d, though PCV2b vaccines demonstrate better efficacy (Opriessnig et al. 2013). Vaccination strategies should account for regional viral variants, as no vaccines currently exist for PCV3 and PCV4 (Rakibuzzaman and Ramamoorthy 2021). Therefore, continued regional viral surveillance is essential for effective vaccine development.

## Conclusion

In conclusion, Porcine circovirus (PCV) significantly impacts the swine industry worldwide, with PCV2 maintaining high prevalence rates in Thailand and PCV3 emerging as a concurrent infection. Despite its widespread circulation, the pathogenicity of PCV3 and the impact of PCV4 remain unclear, necessitating further research and surveillance. In our study, PCV4 was not detected in the upper part of Thailand, particularly in three provinces: Chiang Mai, Lamphun, and Lampang; however, continuous monitoring is recommended. Genetic characterization reveals both regional and global variations in PCV strains, emphasizing the importance of localized epidemiological data for effective vaccine development. While PCV2 vaccines provide partial cross-protection, the absence of vaccines for PCV3 and PCV4 underscores the urgency of developing targeted vaccine strategies to mitigate the economic and health impacts of PCV infections in swine populations.

## Data Availability

No datasets were generated or analysed during the current study.
